# The Ethanol Extract of *Zingiber zerumbet* Attenuates Streptozotocin-Induced Diabetic Nephropathy in Rats

**DOI:** 10.1155/2013/340645

**Published:** 2013-02-14

**Authors:** Thing-Fong Tzeng, Shorong-Shii Liou, Chia Ju Chang, I-Min Liu

**Affiliations:** ^1^Department of Internal Medicine, Pao Chien Hospital, Pingtung City, Pingtung County 90064, Taiwan; ^2^Department of Pharmacy and Graduate Institute of Pharmaceutical Technology, Tajen University, Yanpu Township, Pingtung County 90701, Taiwan; ^3^School of Chinese Pharmaceutical Sciences and Chinese Medicine Resources, China Medical University, Taichung 40402, Taiwan

## Abstract

The ethanol extract from the rhizome of *Zingiber zerumbet* (L.) Smith (EEZZR) has been indicated to possess an insulin-like property by ameliorating hyperglycemia in diabetes. We aimed to investigate whether EEZZR exerts an ameliorative effect on renal damage in diabetes induced by streptozotocin (STZ). Diabetic rats were treated orally with EEZZR (200 and 300 mg kg^−1^ per day) or metformin (100 mg kg^−1^ per day) for 8 weeks. The plasma glucose, creatinine, and blood urea nitrogen as well as urine protein levels and the ratio of kidney weight to body weight were significantly elevated in diabetic rats. EEZZR displayed similar characteristics to those of metformin in reducing hyperglycemia and renal dysfunction in diabetic rats. The histological examinations revealed amelioration of diabetes-induced glomerular pathological changes following the treatment with EEZZR. In addition, the protein expressions of renal nephrin and podocin in diabetic rats were significantly increased following the treatment with EEZZR. The AMP-activated protein kinase (AMPK) protein phosphorylation and expression levels were remarkably reduced in diabetic renal tissues. EEZZR treatment significantly rescued the AMPK phosphorylation compared to nontreated diabetic group. This study suggested that the renoprotective effects of EEZZR may be similar, with the action of metformin, to the prevention of AMPK dephosphorylation and upregulate the expressions of renal nephrin and podocin.

## 1. Introduction

Hyperglycemia and several other symptoms are involved in the development of complications associated with diabetes. Among the complications, diabetic nephropathy (DN) is believed to be the major cause of morbidity and mortality in patients with diabetes and the main cause of the high prevalence of end-stage renal disease in developed countries [1]. It is widely acknowledged that an increase in albumin excretion rate leads to albuminuria and it is the earliest index of DN [2]. The glomerular vasculature consists of three structures that act in concert to prevent the development of albuminuria and proteinuria. These structures are the fenestrated endothelium, the glomerular basement membrane, and the epithelial slit diaphragm [3]. Hyperglycemia leads to a downregulation of negatively charged proteoglycans in the basement membrane of the glomerulus that has been demonstrated [4]. A role of the slit membrane in the pathogenesis of diabetic albuminuria has also been suggested [5]. Amelioration of renal injury resulting in proteinuria in diabetes is reportedly associated with modulating the loss of glomerular permselectivity [6].

*Zingiber zerumbet* (L.) Smith (Zingiberaceae), commonly referred to as pinecone or shampoo ginger, is a perennial, tuberous root herb plant that can be found growing naturally in damp and shaded parts of the lowland or hill slopes, as scattered plants or thickets [7]. Despite its regular uses as a food flavoring and appetizer, *Z. zerumbet *rhizome (ZZR), in particular, has been used traditionally as an herbal medicine in Asian, Indian, Chinese, and Arabic folklores since ancient times [8]. Some traditional uses of ZZR include the treatment of inflammatory- and pain-mediated diseases, worm infestation, and diarrhea [9–11]. Furthermore, the methanol extract of ZZR possesses inhibitory effects on the platelet-activating factor and Den2 virus NS2B/NS3 protease activity [12, 13]. A recent study has shown that the extract of ZZR exerted a potential blood glucose lowering effect in streptozotocin-induced diabetic rats (STZ-diabetic rats) [14]. The ethanol extract of ZZR (EEZZR) resulted in an apparent improvement in the overall insulin sensitivity by ameliorating hyperinsulinemia, and gluconeogenesis has also been demonstrated [15]. ZZR is valued for the ability to promote glucose homeostasis, and it may, therefore, be utilized as an adjuvant therapy in the control of diabetic complications. However, the possibility that ZZR could prove beneficial in the amelioration of diabetic renal damage has not been previously explored. 

The natural history of DN differs according to the type of diabetes and whether microalbuminuria is present. If untreated, 80% of people who have type 1 diabetes and microalbuminuria will progress to overt nephropathy, whereas only 20%–40% of those with type 2 diabetes over a period of 15 years will progress [16]. The present study was conducted to characterize the efficacy of EEZZR on DN of type 1 diabetes utilizing STZ-diabetic rats as an animal model. 

## 2. Materials 

### 2.1. Plant Material and Extraction

ZZR was purchased from a local market in Dongshan, Dongshan District (Tainan City, Taiwan) during October 2010. Macroscopic and microscopic examinations, as well as thin-layer chromatography and high-performance liquid chromatography, were used to confirm the authenticity of the plant material provided (this analysis was performed by Dr. T. Y. Hong, Department of Biotechnology, Collage of Pharmacy and Health Care, Tajen University). Random amplified polymorphic DNA analysis of ZZR supplied was also performed to identify DNA polymorphisms. The voucher specimen (lot no. 20101018) has been deposited in our laboratory. Extraction was performed by maceration and air-dried, and 5 kg of pulverized ZZR was added to 10 L of 95% ethanol at room temperature for 7 days and was occasionally shaken. EEZZR was evaporated to dryness under reduced pressure for the total elimination of alcohol, followed by lyophilization, yielding approximately 575 g of dry residue (w/w yield: 11.5%). EEZZR was kept at –20°C until use and suspended in distilled water. The phytochemical screening of EEZZR revealed the presence of the following classes of chemical compounds: alkaloids, saponins, flavonoids, tannins, terpenoids, phenols, polyphenols, and sugar [17]. The contents of kaempferol, quercetin, curcumin, zerumbone, and 6-gingerol in EEZZR were 266.32 ± 0.21, 82.20 ± 0.14, 75.32 ± 0.18, 200.3 ± 0.37, and 102.5 ± 0.28 *μ*g/g, respectively [15, 17, 18].

### 2.2. Animal Models

Male Wistar rats (8–10 weeks of age, 200–250 g) were obtained from the Animal Center of the National Cheng Kung University Medical College. To induce diabetes, rats were given a single intravenous injection of 60 mg kg^−1^ streptozotocin (STZ; Sigma-Aldrich, Inc., Saint Louis, MO, USA). Animals were considered to be diabetic if they had plasma glucose concentrations of 350 mg dL^−1^ or greater, in addition to polyuria and other diabetic features. All studies were carried out two weeks after the injection of STZ. All animal procedures were performed according to the Guidelines for the Care and Use of Laboratory Animals of the National Institutes of Health (United States), as well as the guidelines of the Animal Welfare Act. These studies were conducted with the approval of the Institutional Animal Care and Use Committee (IACUC) at the Tajen University (approval number: IACUC 99-16; approval date: September 9, 2010).

### 2.3. Treatment Protocols

STZ-diabetic rats were dosed by oral gavage once per day for eight weeks with EEZZR doses of 200 (STZ + EEZZR 200) or 300 mg kg^−1^ (STZ + EEZZR 300) in a volume of 1.5 mL kg^−1^ distilled water. The selection of dosage regime for the present studies was according to the previous report that demonstrated EEZZR at 200 and 300 mg kg^−1^ exerted potential effect in improving insulin resistance in diabetic rats [15]. Another group of STZ-diabetic rats was treated orally for eight weeks with 100 mg kg^−1^ per day metformin (purity ≥ 99.0%, Sigma-Aldrich; STZ + Met). The dose of metformin was based on studies with long-term metformin treatment in STZ-diabetic rats [19]. A vehicle-treated group of STZ-diabetic rats (STZ + vehicle) and normal rats (normal + vehicle) were treated with 1.5 mL kg^−1^ distilled water only over the same treatment period. Body weight, fasting blood glucose, glycosylated hemoglobin (HbA_1c_), and the renal function related parameters were measured every 4 weeks. Urine samples were collected from the mice housed in metabolic cages for 24 h to measure urine volume and urine protein level. 

At the end of the eight-week treatment, rats were sacrificed using an intraperitoneal injection of sodium pentobarbital (50 mg kg^−1^). The kidneys were dissected and rinsed with cold isotonic saline and weighed. Kidney hypertrophy index (KI) was estimated by comparing the wet weight of the kidney to the body weight. The cortical tissues from right kidney were stored immediately at −80°C in liquid nitrogen for biochemical determinations and Western blot analyses. Other kidney tissues were fixed in 10% neutralized formalin for histology.

### 2.4. Blood Sampling and Analysis

Blood samples of rats were centrifuged at 2,000 g for 10 minutes at 4°C; plasma was removed and aliquot for the respective analytical determinations. The diagnostic kit for determinations for plasma levels of glucose (Cat. no. COD12503) was purchased from BioSystem (Barcelona, Spain). The serum creatinine (Cr) concentration was determined by the commercial assay kit (Cat. no. 221-30) purchased from Diagnostic Chemicals Limited (Oxford, CT, USA). Blood urea nitrogen (BUN) was determined by kinetic reagent (Diagnostic Chemicals Limited, Cat. no. 283-30). Commercial enzyme-linked immunosorbent assay (ELISA) kits were used to quantify HbA_1c_ levels (Integrated Bio Ltd., Taipei, Taiwan; Cat. no. CSB-E08140r). All analyses were performed in accordance with the manuals provided by the manufacturers.

### 2.5. Analysis of Urine Parameters

The 24 h urine collected from each diabetic rat and age-matched control was centrifuged at 2,000 g for 10 min. Urinary albumin concentrations were measured by Nephrat II ELISA kit (Cat. no. NR002) obtained from Exocell, INC. (PA, PUA). The creatinine (Cr) concentration in pooled urine samples was determined by the commercial assay kit (Diagnostic Chemicals Limited, Cat. no. 221-30). All analyses were performed in accordance with the manuals provided by the manufacturers. Cr clearance (Ccr) was calculated using the following equation: Ccr (mL min^−1 ^kg body weight) = [urinary Cr (mg dL^−1^) × urinary volume (mL)/serum Cr (mg dL^−1^)] × [1000/body weight (g)] × [1/1440 (min)]. 

### 2.6. Renal Histological Analysis

Renal tissues were fixed with 10% neutral formalin phosphate buffer, dehydrated through a graded alcohol series, embedded in paraffin, cut into 4 *μ*m sections, and stained with periodic acid Schiff (PAS). The sections were examined with light microscopy by an experienced pathologist, and micrographs from six glomeruli were obtained randomly with magnification of 400x. The mean glomerular volume was determined from the mean glomerular capillary tuft area (*A*_*G*_) by light microscopy of PAS sections. The areas were determined by light microscopy and analyzed by dedicated software (Analysis 3.0, Soft Imaging System, Münster, Germany) as the average area of 50 glomerular profiles (the capillary tuft omitting the proximal tubular tissue and Bowman's capsule) for each animal. The glomerular volume (GV) was calculated using the formula GV = *β*/*k*  ×  (*A*_*G*_)^3/2^, where GV is glomerular volume, *β* = 1.38, which is the shape coefficient for spheres (the idealized shape of glomeruli), *k* = 1.1, which is a size distribution coefficient, and *A*_*G*_ is the glomerular capillary tuft area [20]. The index of mesangial expansion was scored by a quantitative estimate of the mesangial zones width in each glomerulus, expressed as a function of the total glomerular area [21]: 0: normal glomeruli; 1: matrix expansion occurred in up to 50% of a glomerulus; 2: matrix expansion occurred in 50%–75% of a glomerulus; 3: matrix expansion occurred in 75%–100% of a glomerulus.

### 2.7. Western Blot Analysis

Renal cortical sections were homogenized with ice-cold lysis buffer (pH 7.5) containing 137 mmol L^−1^ NaCl, 20 mmol L^−1^ Tris-HCl, 1% Tween 20, 10% glycerol, 1 mmol L^−1^ PMSF, and protease inhibitor mixture dimethyl sulfoxide solution. The homogenates were centrifuged at 2000 ×g at 4°C. Before immunoblotting, and the protein concentration of each tissue was determined using a Bio-Rad protein assay kit (Bio-Rad Laboratories, Japan) and bovine serum albumin as a standard, to ensure equal loading among lanes. An aliquot of protein (50 *μ*g) from each sample was electrophoresed through 8% and 12% sodium dodecyl sulfate polyacrylamide gels, and then separated protein was transferred at 4°C onto a polyvinylidene difluoride membrane (Santa Cruz Biotechnology, Inc.). The membrane was blocked with 5% skimmed milk in TBS (150 mmol L^−1^ NaCl, 0.01 mmol L^−1^ Tris-HCl, pH 7.6) for 2 h and then incubated with primary antibodies against nephrin (Santa Cruz Biotechnology, Inc., Cat. no. sc-28192), podocin (Santa Cruz Biotechnology, Inc., Cat. no. sc-21009), AMP-activated protein kinase (AMPK)*α* (Cell Signaling Technology; Cat. no. 2352), phospho-AMPK*α* at Thr 172 (pAMPK*α*; Cell Signaling Technology; Cat. no. 2531), or *β*-actin (Santa Cruz Biotechnology, Inc.; Cat. no. sc-130656), respectively, overnight at 4°C according to the manufacturer's instructions. After three 5 min washes in Tris-buffered saline with Tween 20 (TBST) (20 mmol L^−1^ Tris-HCl, pH 7.5, 150 mmol L^−1^ NaCl, and 0.05% Tween 20), membranes were incubated with the appropriate peroxidase-conjugated secondary antibodies. The membranes were then washed 3 times in TBST and visualized on X-ray film using the ECL advance Western Blotting detection kit (Cat. no. RPN2135; GE Healthcare Life Sciences, NJ, USA). Band densities were determined using ATTO Densitograph Software (ATTO Corporation, Tokyo, Japan) and quantified as the ratio to *β*-actin. Renal cortical sections were sampled from 4 independent experiments. All experimental sample values were then expressed relative to this adjusted mean value. 

### 2.8. Statistical Analysis

The results are presented as the mean ± standard deviation (SD) for each group of animals at the number (*n*) indicated. Statistical analysis was performed with one-way analysis of variance (ANOVA). The Dunnett range post hoc comparisons were used to determine the source of significant differences where appropriate. The renal morphohistology and the morphologic analysis for PAS staining were analyzed statistically using the Kruskal-Wallis test and Dunn's multiple comparisons test. Values of *P* < .05 were considered statistically significant.

## 3. Results

### 3.1. Body Weight, Fasting Blood Glucose, and Glycosylated Hemoglobin Content

During 8-week experiment, STZ-diabetic rats were found to have significant weight loss when compared with normal rats. The body weight reduction was not obvious in STZ-diabetic rats receiving EEZZR or metformin during the experimental period. A significant increase in fasting blood glucose in STZ-diabetic rats was observed when compared to normal control group and this change was more marked at the 8th week following diabetes induction. Furthermore, the blood glucose lowering effect was obvious when STZ-diabetic rats were treated with 300 mg kg^−1^ EEZZR (28.1 ± 2.3%) and metformin (38.6 ± 4.3%) for 8 weeks (Table 1).

The value of HbA_1c_ was markedly higher in STZ-diabetic rats when compared with normal rats (Table 1). Treatment with 300 mg kg^−1^ ZZREE or metformin for 8 weeks decreased the levels of HbA_1c_ in STZ-diabetic rats by 24.6 ± 2.6% and 34.4 ± 3.2% relative to the value in STZ-diabetic rats that received vehicle, respectively (Table 1).

### 3.2. Renal Function Related Parameters

During the experiment period, STZ-diabetic rats showed an increase in 24 h urine volume, accompanied by an increase in urine protein excretion. After 8 weeks of EEZZR treatment, 24 h urine volume and 24 h urine protein excretion of STZ-diabetic rats were markedly less than their vehicle-treated counterparts (Table 2). In addition, the levels of Scr and BUN in STZ-diabetic rats were obviously higher than those of normal control group, and there was an effective reduction in the levels of Scr and BUN in STZ-diabetic rats receiving 8 weeks of EEZZR or metformin treatment when compared with their vehicle counterparts (Table 2). In particular, after EEZZR or metformin treatment, increased Ccr in STZ-diabetic rats was observed (Table 2). 

The mean kidney weight and the ratio of kidney weight to body weight in vehicle-treated STZ-diabetic rats were significantly increased as compared to those in the normal control group (Table 3). Treatment of STZ-diabetic rats with EEZZR or metformin obviously ameliorated kidney hypertrophy index (Table 3).

### 3.3. Renal Histology

**Figure 1:**
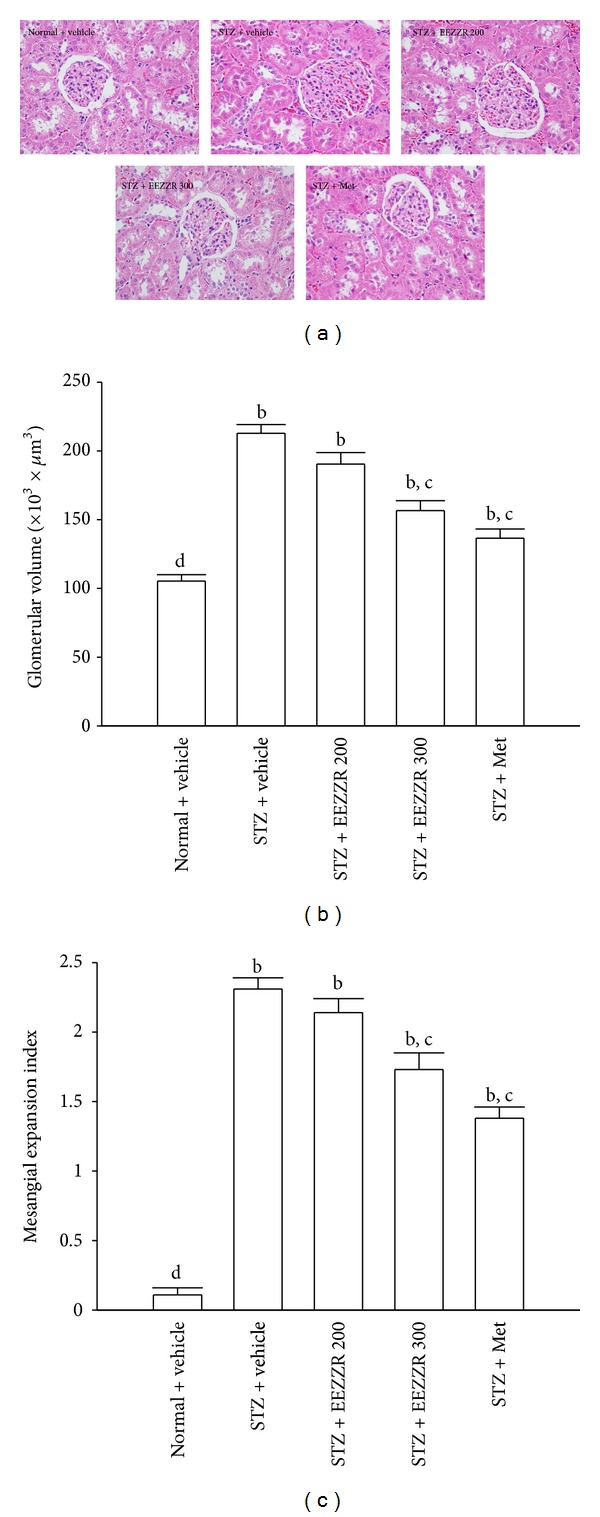
(a) Representative photomicrographs (original magnification, 400x) of PAS-stained kidney sections, (b) glomerular volume, and (c) expansion of matrix index expressed as a quantitative estimate score in experimental groups. STZ-diabetic rats were dosed by oral gavage once per day for eight weeks with 100 mg kg^−1^  per day metformin (STZ + Met), 200 mg kg^−1^ per day EEZZR (STZ + EEZZR 200), or 300 mg kg^−1^ EEZZR (STZ + EEZZR 300). Normal or STZ-diabetic rats receiving vehicle treatment were given the same volume of vehicle (distilled water) used to dissolve the test medications. Values (mean ± SD) were obtained for each group of 4 animals. ^b^ *P* < .01 compared to the values of vehicle-treated normal rats (normal + vehicle). ^c^ *P* < .05 and ^d^ *P* < .01 compared to the values of vehicle-treated STZ-diabetic rats (STZ + vehicle), respectively.

Histological examination of the kidneys through light microscope showed that glomerular hypertrophy and expansion of the mesangial area were induced by STZ (Figure 1(a)). Morphometric analysis indicated that treatment or metformin for 8 weeks significantly inhibited the increase of glomerular volume in STZ-diabetic rats compared with vehicle treatment (Figure 1(b)). After 8-week treatment with EEZZR or metformin, glomerular hypertrophy and the increase of mesangial expansion index induced by STZ were significantly inhibited compared with those of vehicle-treated rats (Figure 1(c)). 

### 3.4. Changes in Protein Expressions of Nephrin and Podocin

**Figure 2:**
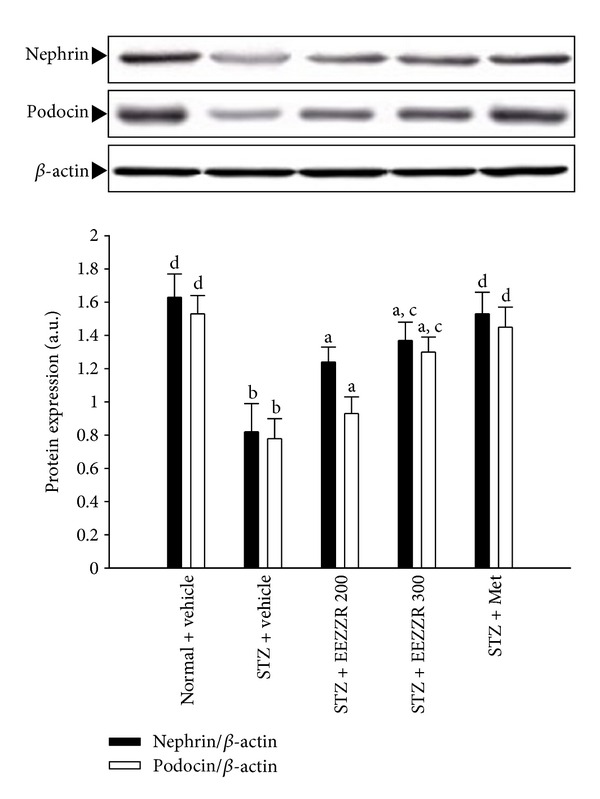
Effects of treatments on protein expressions of renal nephrin and podocin in the renal tissues of STZ-diabetic rats. STZ-diabetic rats were dosed by oral gavage once per day for eight weeks with 100 mg kg^−1^ per day metformin (STZ + Met), 200 mg kg^−1^ per day EEZZR (STZ + EEZZR 200), or 300 mg kg^−1^ EEZZR (STZ + EEZZR 300). Normal or STZ-diabetic rats receiving vehicle treatment were given the same volume of vehicle (distilled water) used to dissolve the test medications. Values (mean ± SD) were obtained for each group of 4 animals. ^a^ *P* < .05 and ^b^ *P* < .01 compared to the values of vehicle-treated normal rats (normal + vehicle). ^c^ *P* < .05 and ^d^ *P* < .01 compared to the values of vehicle-treated STZ-diabetic rats (STZ + vehicle), respectively.

Western blot assay indicated that the renal nephrin and podocin proteins were less expressed in vehicle-treated STZ-diabetic rats (Figure 2). Eight weeks of EEZZR or metformin treatment partially prevented diabetes-induced loss of glomerular nephrin and podocin expressions (Figure 2).

### 3.5. Protein Expression and Phosphorylation of AMPK

**Figure 3:**
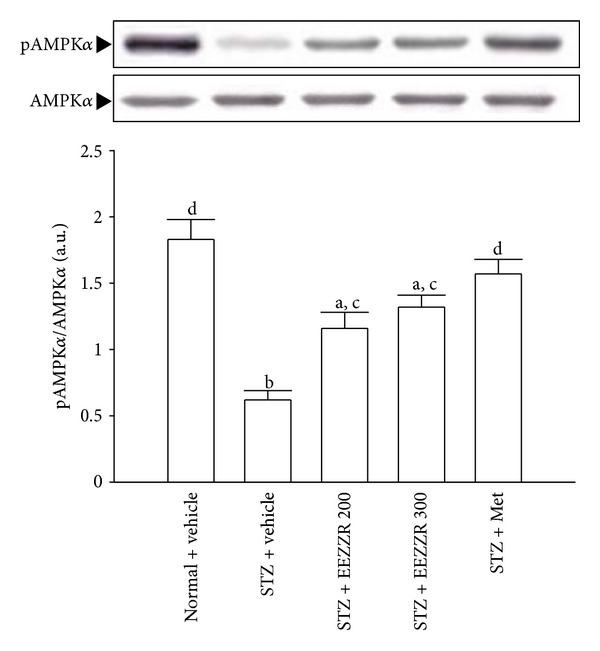
Effects of treatments on protein expression and phosphorylation of AMPK in the renal tissues of STZ-diabetic rats. STZ-diabetic rats were dosed by oral gavage once per day for eight weeks with 100 mg kg^−1^ per day metformin (STZ + Met), 200 mg kg^−1^ per day EEZZR (STZ + EEZZR 200), or 300 mg kg^−1^ EEZZR (STZ + EEZZR 300). Normal or STZ-diabetic rats receiving vehicle treatment were given the same volume of vehicle (distilled water) used to dissolve the test medications. Values (mean ± SD) were obtained for each group of 4 animals. ^a^ *P* < .05 and ^d^ *P* < .01 compared to the values of vehicle-treated normal rats (normal + vehicle). ^c^ *P* < .05 and ^d^ *P* < .01 compared to the values of vehicle-treated STZ-diabetic rats (STZ + vehicle), respectively.

The immunoblot results showed that the STZ led to a decrease in the phosphorylation degree of AMPK in kidney as compared to the normal rats (Figure 3). However, there was no difference in the protein levels of AMPK between any groups. The STZ significantly reduced the pAMPK/AMPK ratio (by 33.8% relative to those in vehicle-treated normal rats, *P* < .01) in the kidneys of the rats (Figure 3). These STZ-induced downregulations in the ratio of pAMPK/AMPK were significantly reversed in the kidney after 8-week treatment with EEZZR (300 mg kg^−1^ per day) by 1.2-fold increases relative to those in vehicle-treated STZ-diabetic rats (*P* < .05, Figure 3). Treatment STZ-diabetic rats with metformin also significantly upregulated the ratio of pAMPK/AMPK in the kidney to 2.5-fold relative to that in vehicle-treated STZ-diabetic rats (*P* < .05, Figure 3). 

## 4. Discussion

It has reported a reduction in the apparent podocyte number and podocyte density as early as two weeks after STZ injection, an effect that appeared to worsen somewhat at six weeks and even further at eight weeks [22]. Urinary albumin excretion has been demonstrated to be a good clinical predictor of renal lesions in DN [2]. In the present study, the increase in urinary albumin concentration corresponding to hyperglycemia was more pronounced eight weeks following the induction of diabetes. In addition, serum Cr and BUN levels and creatinine clearance, generally considered as markers of renal function, were higher in STZ-diabetic rats than those of nondiabetic group. Histological examination of the kidneys through light microscope manifested that glomerular hypertrophy and mesangial matrix accumulation were induced in the diabetic. These results demonstrated that the diabetic renal injury model presenting renal hypertrophy, renal glomerular damage, and renal dysfunction was successfully created.

After 8-week treatment, we found that EEZZR lowered fasting blood glucose and HbA_1c_ of diabetic rats, similar to the effect of metformin. BUN, Scr, urine volume, and proteinuria over 24 h were effectively reduced in EEZZR-treated diabetic rats compared to diabetic control group. In this study, both ZZREE and metformin caused significant improvements in the relative kidney weight, suggesting that they may reverse the hypertrophy in STZ-diabetic rats. In addition, EEZZR ameliorated glomerular pathological changes in diabetic kidney similar to the effects produced by metformin. Metformin exerts an insulin-sensitizing effect through the activation of the AMPK pathway [23]. Although the effects of metformin were more effective than those produced by EEZZR, we concededly demonstrated that EEZZR attenuated DN syndrome characterized by proteinuria and the loss of renal function in STZ-diabetic rats.

As the final and major size barrier to the passage of proteins and other macromolecules, slit diaphragm, the key structure of foot process, plays a crucial role in the occurrence and development of proteinuria [3]. Nephrin, a transmembrane protein belonging to the immunoglobulin superfamily of cell adhesion molecules, was the first identified structural protein of the podocyte slit diaphragm which has a dramatic functional importance [24]. Attenuation of nephrin expression in experimental kidney diseases is associated with a loss of the slit diaphragm and massive proteinuria [24]. Podocin, a stomatin family member, is another important component of the glomerular slit diaphragm complex which colocalizes and interacts with cytosolic tail of nephrin in the lipid rafts of the podocyte foot process cell membrane [25]. Mutations in the podocin gene cause severe structural podocyte alterations and massive proteinuria leading to nephrotic syndrome [25]. It has been demonstrated that the reduction of podocin leads to decreased expression and obvious redistribution of nephrin [26]. Our observations demonstrated that rats with diabetes induced by STZ lost their functional podocin, exhibited less nephrin protein expressions, and developed proteinuria. This downregulated expression of renal nephrin and podocin protein was alleviated in the 8-week treatment with EEZZR or metformin, which could be a main contributor reducing proteinuria in STZ-diabetic rats.

AMPK is highly expressed in the kidney where it is reported to be involved in a variety of physiological and pathological processes including ion transport, podocyte function, and diabetic renal hypertrophy [27]. Thus, attention has been drawn to the modulation of AMPK signal transduction to attenuate diabetes-affected renal dysfunction. Ready availability of agents such as metformin and thiazolidinediones, which increase AMPK activity, makes AMPK an attractive therapeutic target for reversing diabetes-induced renal injury [28]. We observed that EEZZR poses the similar effect of metformin that could prevent renal AMPK dephosphorylation in insulin-deficient diabetic rats. Therefore, we suggest that EEZZR has protective effects on several pharmacological targets in DN. 

It was demonstrated that the suppression of AMPK was interposed by hyperglycemia [28]. Decreased phosphorylation of AMPK was contributed to hyperglycemia-associated renal injury. It should be noted that the significant influence of EEZZR on hyperglycemia in STZ-diabetic rats was observed, which suggested that the renal protective effect of EEZZR might be also related to plasma glucose lowering pathway(s). Whether this is a direct stimulatory effect of AMPK or if other intracellular pathways are involved in the action of EEZZR has to be ruled out in the future studies. 

EEZZR has been the subject of extensive chemical investigations due to their high medicinal values. The chemical constituents that are more frequently found in EEZZR are flavonoids, such as kaempferol, quercetin, and curcumin [15, 17, 18]. The volatile oils of EEZZR have been reported to contain a cyclic sesquiterpene zerumbone or 2,6,9-humulatrien-8-one as the major component, as well as humulene and camphene [8]. The specific components of EEZZR mainly responsible for the protective effect on renal are to be identified in the future research work.

## 5. Conclusion

The results obtained in the present study suggest that EEZZR ameliorate STZ-induced proteinuria and kidney injury in rats at least in part, by prevention of AMPK dephosphorylation, modulating slit diaphragm associated molecular nephrin and podocin expressions. This study provides an important pharmacological and therapeutic basis for the treatment of kidney diseases in the insulin-deficient diabetes.

## Figures and Tables

**Table 1:** Effects of different treatments on body weight, fasting blood glucose, and HbA_1c_ levels in rats.

Groups	Week 4	Week 8
Body weight (g rat^−1^)		
Normal + vehicle	291.13 ± 14.13^d^	341.64 ± 13.48^d^
STZ + vehicle	183.98 ± 13.14^b^	221.28 ± 15.93^b^
STZ + EEZZR 200	216.73 ± 12.26^b,c^	242.62 ± 14.27^b,c^
STZ + EEZZR 300	254.32 ± 15.09^a,c^	282.71 ± 16.45^a,c^
STZ + Met	276.85 ± 11.23^d^	318.23 ± 13.21^d^
Plasma glucose (mg dL^−1^)		
Normal + vehicle	93.42 ± 6.13^d^	95.62 ± 7.83^d^
STZ + vehicle	383.12 ± 15.26^b^	401.53 ± 17.92^b^
STZ + EEZZR 200	332.29 ± 14.16^b,c^	317.07 ± 15.36^b,c^
STZ + EEZZR 300	314.56 ± 16.29^b,c^	289.42 ± 16.58^b,c^
STZ + Met	273.46 ± 13.78^b,d^	237.39 ± 14.26^b,d^
HbAlc (%)		
Normal + vehicle	4.81 ± 1.03^d^	4.87 ± 1.21^d^
STZ + vehicle	13.09 ± 2.67^b^	13.62 ± 3.26^b^
STZ + EEZZR 200	11.63 ± 1.75^b^	11.21 ± 1.74^b^
STZ + EEZZR 300	11.12 ± 3.07^b^	10.41 ± 2.92^b^
STZ + Met	9.96 ± 2.23^b^	8.93 ± 2.17^b^

STZ-diabetic rats were dosed by oral gavage once per day for eight weeks with 100 mg kg^−1^ per day metformin (STZ + Met), 200 mg kg^−1^ per day EEZZR (STZ + EEZZR 200), or 300 mg kg^−1^ EEZZR (STZ + EEZZR 300). Normal or STZ-diabetic rats receiving vehicle treatment were given the same volume of vehicle (distilled water) used to dissolve the test medications. Values (mean ± SD) were obtained for each group of 8 animals. ^a^ *P* < .05 and ^b^ *P* < .01 compared to the values of vehicle-treated normal rats (normal + vehicle). ^c^ *P* < .05 and ^d^ *P* < .01 compared to the values of vehicle-treated STZ-diabetic rats (STZ + vehicle), respectively.

**Table 2:** Effects of different treatments on renal function related parameters in rats.

Groups	Week 4	Week 8
Urine volume (mL 24 h^−1^)		
Normal + vehicle	5.37 ± 3.28^d^	8.99 ± 3.92^d^
STZ + vehicle	26.14 ± 9.46^b^	27.34 ± 9.01^b^
STZ + EEZZR 200	18.23 ± 10.27^b^	17.51 ± 7.83^b^
STZ + EEZZR 300	14.89 ± 7.09^b^	15.24 ± 8.74^b^
STZ + Met	13.03 ± 8.17^b^	12.29 ± 6.43^b^
Urine protein (mg 24 h^−1^)		
Normal + vehicle	6.13 ± 3.62^d^	6.57 ± 3.17^d^
STZ + vehicle	26.59 ± 5.17^b^	30.17 ± 7.26^b^
STZ + EEZZR 200	18.24 ± 6.32^a,c^	15.79 ± 5.23^a,c^
STZ + EEZZR 300	12.98 ± 4.01^d^	11.81 ± 3.17^d^
STZ + Met	10.13 ± 5.33^d^	8.86 ± 4.06^d^
Serum Cr (μmol L^−1^)		
Normal + vehicle	38.26 ± 6.38^d^	40.13 ± 7.14^d^
STZ + vehicle	71.59 ± 8.45^b^	100.36 ± 10.21^b^
STZ + EEZZR 200	67.43 ± 9.08^b^	89.11 ± 8.37^b^
STZ + EEZZR 300	58.21 ± 8.23^a,d^	72.23 ± 9.25^a,d^
STZ + Met	49.76 ± 8.12^a,d^	56.24 ± 8.76^a,d^
BUN (mmol L^−1^)		
Normal + vehicle	6.47 ± 1.92^c^	7.22 ± 1.39^c^
STZ + vehicle	12.76 ± 2.94^a^	16.31 ± 3.02^a^
STZ + EEZZR 200	10.38 ± 2.41^a^	12.19 ± 2.33^a^
STZ + EEZZR 300	8.46 ± 1.71	11.47 ± 2.26
STZ + Met	7.32 ± 1.83	10.18 ± 2.14
Ccr (mL min^−1^ per kg)		
Normal + vehicle	3.68 ± 1.28	3.82 ± 0.96^a^
STZ + vehicle	1.86 ± 1.04	1.73 ± 0.92^c^
STZ + EEZZR 200	2.43 ± 0.84	2.22 ± 0.78
STZ + EEZZR 300	2.85 ± 0.52	2.64 ± 1.24
STZ + Met	3.12 ± 0.73	3.16 ± 0.92

STZ-diabetic rats were dosed by oral gavage once per day for eight weeks with 100 mg kg^−1^ per day metformin (STZ + Met), 200 mg kg^−1^ per day EEZZR (STZ + EEZZR 200), or 300 mg kg^−1^ EEZZR (STZ + EEZZR 300). Normal or STZ-diabetic rats receiving vehicle treatment were given the same volume of vehicle (distilled water) used to dissolve the test medications. Values (mean ± SD) were obtained for each group of 8 animals. ^a^ *P* < .05 and ^b^ *P* < .01 compared to the values of vehicle-treated normal rats (normal + vehicle). ^c^ *P* < .05 and ^d^ *P* < .01 compared to the values of vehicle-treated STZ-diabetic rats (STZ + vehicle), respectively.

**Table 3:** Effects of different treatments on kidney weight and kidney hypertrophy index (KI) in rats at the end of the eight-week treatment.

Groups	Kidney weight (g)	KI (mg g^−1^)
Normal + vehicle	1.3 ± 0.2^d^	3.8 ± 1.2^d^
STZ + vehicle	2.7 ± 0.2^b^	12.2 ± 3.6^b^
STZ + EEZZR 200	2.2 ± 0.4^b^	8.3 ± 2.8
STZ + EEZZR 300	2.2 ± 0.3^b^	7.2 ± 1.9^a^
STZ + Met	1.8 ± 0.4^c^	5.5 ± 1.7^c^

STZ-diabetic rats were dosed by oral gavage once per day for eight weeks with 100 mg kg^−1^ per day metformin (STZ + Met), 200 mg kg^−1^ per day EEZZR (STZ + EEZZR 200), or 300 mg kg^−1^ EEZZR (STZ + EEZZR 300). Normal or STZ-diabetic rats receiving vehicle treatment were given the same volume of vehicle (distilled water) used to dissolve the test medications. Values (mean ± SD) were obtained for each group of 8 animals. ^a^ *P* < .05 and ^b^ *P* < .01 compared to the values of vehicle-treated normal rats (normal + vehicle). ^c^ *P* < .05 and ^d^ *P* < .01 compared to the values of vehicle-treated STZ-diabetic rats (STZ + vehicle), respectively.
